# Colicin F_Y_ inhibits pathogenic *Yersinia enterocolitica* in mice

**DOI:** 10.1038/s41598-018-30729-7

**Published:** 2018-08-16

**Authors:** Juraj Bosák, Lenka Micenková, Matěj Hrala, Katarína Pomorská, Michaela Kunova Bosakova, Pavel Krejci, Eduard Göpfert, Martin Faldyna, David Šmajs

**Affiliations:** 1 Department of Biology, Faculty of Medicine, Masaryk University, Brno, Czech Republic; 2 Research Centre for Toxic Compounds in the Environment, Faculty of Science, Masaryk University, Brno, Czech Republic; 3 Veterinary Research Institute, Brno, Czech Republic

## Abstract

Yersiniosis belongs to the common foodborne diseases around the world, and frequently manifests as diarrhea that can be treated with probiotics. Colicin F_Y_ is an antibacterial agent produced by bacteria and it is capable of specific growth inhibition of *Yersinia enterocolitica*, the causative agent of gastrointestinal yersiniosis. In this study, recombinant *E*. *coli* producing colicin F_Y_ were constructed, using both known probiotic strains EcH22 and EcColinfant, and the newly isolated murine strains Ec1127 and Ec1145. All *E*. *coli* strains producing colicin F_Y_ inhibited growth of pathogenic *Y*. *enterocolitica* during co-cultivation *in vitro*. In dysbiotic mice treated with streptomycin, *E*. *coli* strains producing colicin F_Y_ inhibited progression of *Y*. *enterocolitica* infections. This growth inhibition was not observed in mice with normal gut microflora, likely due to insufficient colonization capacity of *E*. *coli* strains and/or due to spatial differences in intestinal niches. Isogenic *Y*. *enterocolitica* producing colicin F_Y_ was constructed and shown to inhibit pathogenic *Y*. *enterocolitica* in mice with normal microflora. Evidence of *in vivo* antimicrobial activity of colicin F_Y_ may have utility in the treatment of *Y*. *enterocolitica* infections.

## Introduction

A total of 6,861 confirmed cases of yersiniosis (i.e., infections caused by *Yersinia enterocolitica*) were reported in the European Union in 2016, making yersiniosis the third most common human zoonosis in the EU^1^. In the United States, *Yersinia enterocolitica* causes an estimated 100,000 infections annually^2,3^. Infections caused by *Y*. *enterocolitica* range from self-limited enteritis to life-threatening systemic infections, however, the most common manifestation is diarrhea, and occurs mainly in children^4–7^. Several studies also support the idea that *Y*. *enterocolitica* infection may be associated with the development of chronic inflammatory bowel diseases^8^. Although antibiotic treatment is recommended for serious cases, the benefits of antibiotic therapy in uncomplicated cases have not been well demonstrated^9,10^. Instead, rehydration and use of probiotics are often suggested for simple diarrheal cases.

Probiotics are live microorganisms that confer a health benefit on the host when administrated in adequate amounts^11^. Many probiotic products are based on particular strains of lactic acid bacteria, such as *Lactobacillus*, *Lactococcus*, or *Bifidobacterium* species; however, *Escherichia coli* and other bacteria (and even yeast species) have been used as probiotics^12^. Among several probiotic strains patented for commercial applications, *E*. *coli* strains are part of three approved human probiotic drugs: Mutaflor (Ardeypharm GmbH; Herdecke, Germany), Symbioflor-2 (Symbiopharm GmbH; Herborn, Germany), and Colinfant New Born (Dyntec; Terezín, Czech Republic)^12^.

Production of antimicrobial substances is one of the most important features in the context of bacterial fitness and also in terms of probiotic efficacy^13^. Bacteriocins are antimicrobial peptides or proteins produced by many bacterial species, including probiotic strains, and bacteriocin preparations have been successfully used in food preservation and in veterinary medicine (reviewed in^14,15^). In the *Enterobacteriaceae* family, bacteriocins are frequently produced by *E*. *coli* strains^16,17^. Among *E*. *coli*, two molecular types of bacteriocins have been described, including microcins (peptides) and colicins (proteins). To date, more than twenty colicins have been characterized in various levels of detail (reviewed in^18,19^) and several colicin types have been shown to specifically inhibit pathogenic bacteria *in vitro*^20–25^. One of the well-characterized colicins, colicin F_Y_, is produced by *Yersinia frederiksenii* Y27601, which harbors a plasmid with colicin F_Y_ activity and immunity genes (*cfy*A and *cfy*I, respectively). Besides the *in vitro* activity against several nonpathogenic and opportunistic yersiniae (i.e., *Y*. *frederiksenii*, *Y*. *aldovae*, *Y*. *kristensenii*, and *Y*. *intermedia*), colicin F_Y_ is also very effective against *Y*. *enterocolitica*, the causative agent of gastrointestinal yersiniosis. The exceptionally wide susceptibility of *Y*. *enterocolitica* strains to colicin F_Y_, together with an absence of activity towards bacterial strains outside the *Yersinia* genus, is a consequence of the interaction between colicin F_Y_ and the yersinia-specific outer membrane protein YiuR, which also serves as a colicin F_Y_ receptor molecule^20,21^.

In this study, recombinant probiotic *E*. *coli* strains, producing colicin F_Y_, were constructed and their therapeutic potential against *Y*. *enterocolitica* was analyzed *in vitro* and *in vivo*. The *in vivo* activity of colicin F_Y_ was also studied using isogenic and recombinant colicin F_Y_ producing *Yersinia* strain.

## Results

### *Escherichia coli* strains and their intestinal colonization capacity

**Figure 1:**
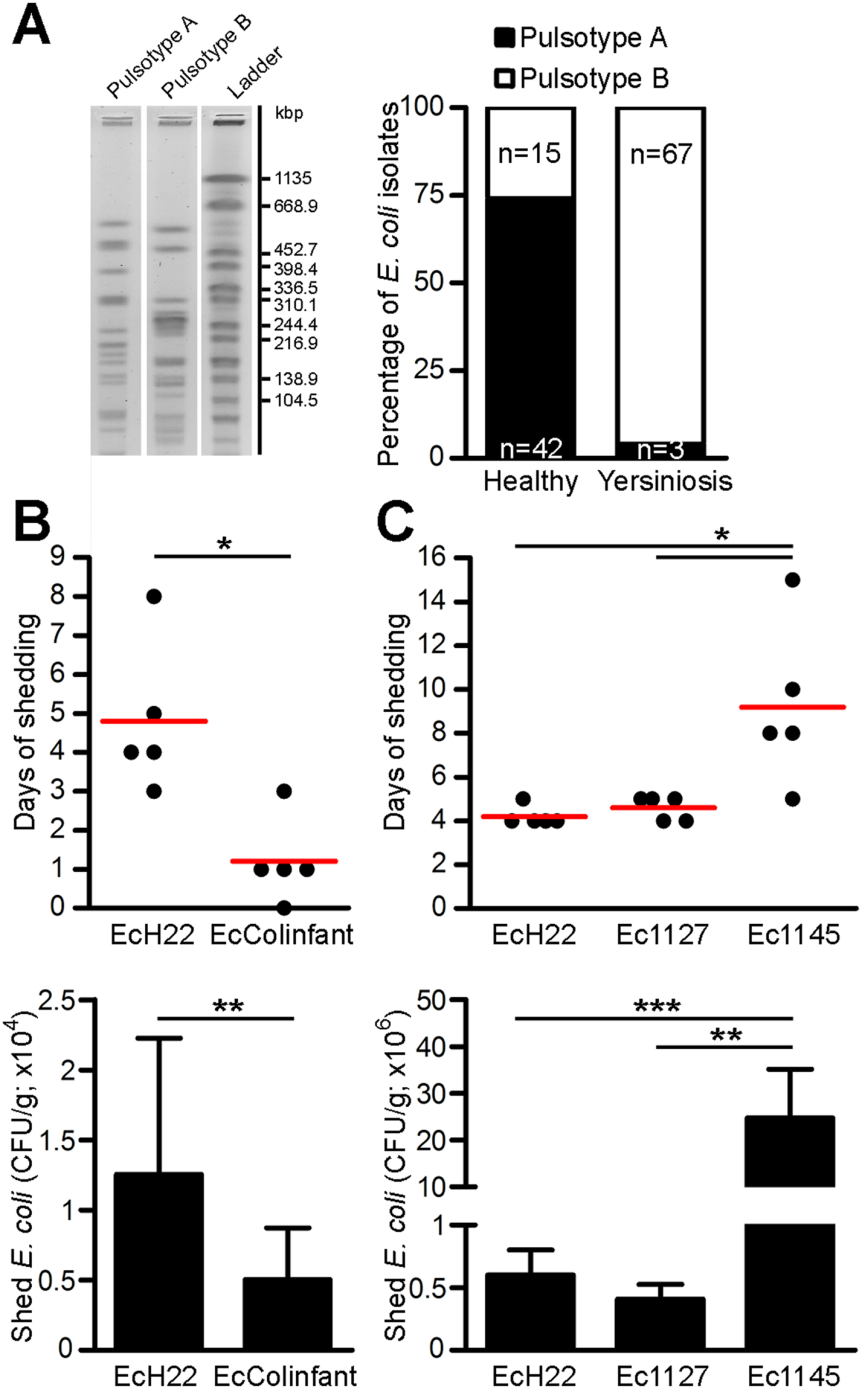
Characterization of murine *E*. *coli* isolates and colonization capacity of recombinant *E*. *coli* strains producing colicin F_Y_. (**A**) Feces from four healthy mice and five mice with yersiniosis were collected for five days. A set of 127 *E*. *coli* isolates was obtained and resolved using PFGE, which found two pulsotypes. Strain Ec1127 was predominant in mice with yersiniosis, while strain Ec1145 was frequently isolated from healthy mice. The original gel is in Supplementary Fig. S1. (**B,C**) Mice (n = 5; each group) were inoculated with 10^7^ CFU of *E*. *coli* and the fecal counts of recombinant *E*. *coli* were monitored for 15 days. The duration of shedding (top; red bar, mean) and the numbers of shed *E*. *coli* during the first five days (bottom; mean ± SEM) were plotted. EcH22 showed longer and stronger colonization capacity than EcColinfant (**B**). While Ec1127 showed colonization comparable to EcH22, Ec1145 displayed superior length and strength relative to *E*. *coli* shedding (**C**). The end of colonization was defined as two consecutive days without shedding of recombinant *E*. *coli*. Two-tailed Mann–Whitney–U test was used for statistical comparisons (*p < 0.05, **p < 0.01, ***p < 0.001). Raw data for colonization capacity of *E*. *coli* strains are shown in Supplementary Fig. S1.

Since human *E*. *coli* strains are weak colonizers of the murine gut^26,27^, murine *E*. *coli* strains were isolated and used in this study. Among 127 murine *E*. *coli* isolates, pulsed field gel electrophoresis (PFGE) analysis found only two *E*. *coli* pulsotypes (Fig. 1A; see Methods). While strain Ec1127 was the dominant *E*. *coli* in mice with yersiniosis, strain Ec1145 was frequently isolated from healthy controls. In addition to Ec1127 and Ec1145, two *E*. *coli* strains with known probiotic features (EcH22 and EcColinfant) were used^12,28^.

All four *E*. *coli* strains were transformed to stably maintain the recombinant colicinogenic plasmid and then tested for their capacity to colonize the murine gastrointestinal tract. While human EcH22 and murine Ec1127 showed similar colonization capacities, the colonization capacity of EcColinfant was considerably lower (*p < 0.05). EcColinfant was not detectable after day three of the experiment and lower amounts of bacteria were shed compared to EcH22 (**p < 0.01; Figs 1B and S1). The best colonization capacity was observed for murine Ec1145, which was detected up to fifteen days post infection (*p < 0.05) and the number of bacteria in mice feces during the first five days was approximately 40-times higher than for EcH22 and Ec1127 (***p < 0.001 and **p = 0.001, respectively; Figs 1C and S1). In addition, recombinant colicin producers showed similar colonization capacity compared to isogenic nonproducers (Supplementary Fig. S2).

### Recombinant expression and intestinal stability of colicin F_Y_

**Figure 2:**
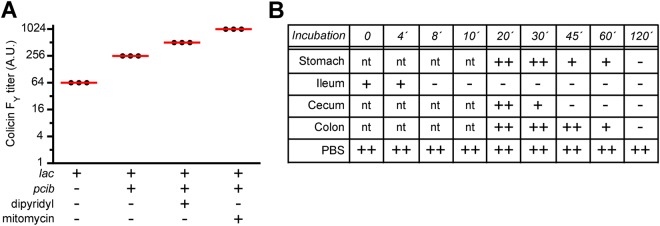
Recombinant expression and stability of colicin F_Y_ under gastrointestinal conditions. (**A**) Colicin F_Y_ expression from the *lac* promoter alone, or in combination with *pcib* promoter was analyzed *in vitro* by spotting dilutions of colicin F_Y_ extracts on agar plates with susceptible *Yersinia*. The *pcib* promoter enhanced colicin F_Y_ expression and allowed the inducible expression of colicin F_Y_ by iron limitation (dipyridyl) and the SOS response (mitomycin). Three independent experiments are shown (red bar, mean). A.U. (arbitrary unit) – an inverted value of the highest dilution of crude colicin extract causing growth inhibition. (**B**) Colicin F_Y_ extract was incubated with murine gastrointestinal fractions, and the residual activity of colicin F_Y_, at various time-points, was measured by spotting diluted suspensions on agar plates with susceptible *Yersinia*. Colicin F_Y_ stayed active for more than forty-five minutes in the colon contents. A representative result from three independent experiments is shown. ^++^activity of 10-fold diluted colicin F_Y_; ^+^activity of non-diluted colicin F_Y_; ^−^undetectable activity; nt, not tested.

Recombinant expression of colicin F_Y_ was tested *in vitro* using two recombinant expression systems, i.e., one with the basal *lac* expression of colicin F_Y_ and the second was with colicin F_Y_ expression controlled by the *lac* promoter and also by the gut inflammation-dependent promoter of colicin Ib (*pcib*) (constructs pDS1006 and pDS1281, respectively; see Methods). The presence of the *pcib* promoter enhanced colicin F_Y_ expression compared to expression from the *lac* promoter alone; moreover, colicin F_Y_ expression was inducible with iron limitation and the SOS response *in vitro* (Fig. 2A). Colicin F_Y_ recombinant expression controlled by both promoters was used throughout this study.

To analyze colicin activity under gastrointestinal tract conditions, the contents of murine stomach, ileum, cecum, and colon were collected. Colicin F_Y_ was incubated with gastrointestinal fractions and its activity was analyzed over time. While ileum contents inactivated colicin F_Y_ within a few minutes, colicin F_Y_ stayed active for more than 45 minutes when cultivated with the colon contents (Fig. 2B).

### *In vitro* activity of recombinant colicinogenic *E*. *coli* against pathogenic *Y*. *enterocolitica*

**Figure 3:**
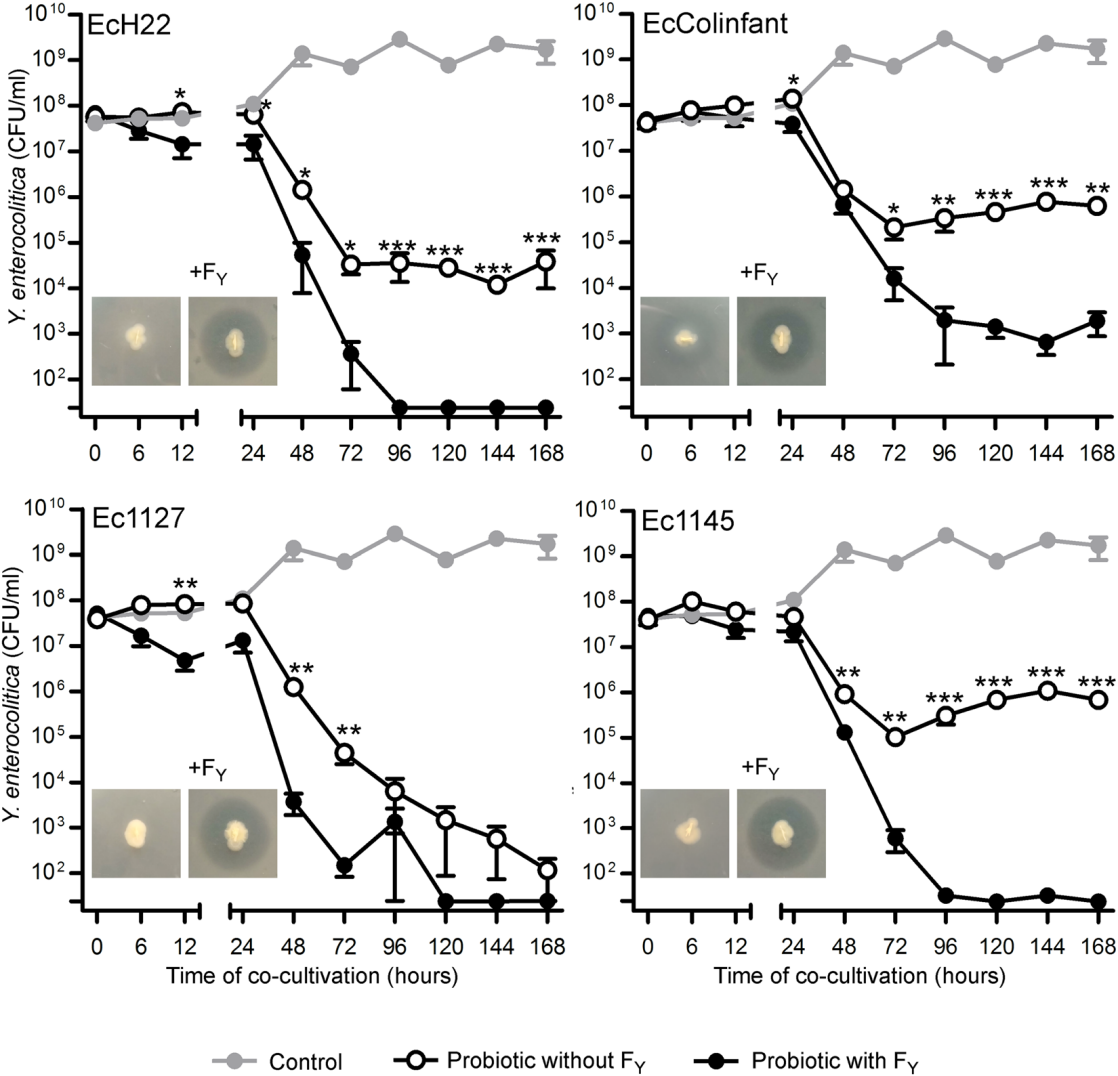
*In vitro* activity of colicin F_Y_-producing *E*. *coli* strains against pathogenic *Y*. *enterocolitica*. *Y*. *enterocolitica* was co-cultivated (37 °C, 200 rpm) with recombinant *E*. *coli* strains that either produced or did not produce colicin F_Y_. Numbers of *Y*. *enterocolitica* were counted at various time-points and plotted (mean ± SEM). Production of colicin F_Y_ enhanced inhibition activity of the *E*. *coli* strains. *Y*. *enterocolitica* was eliminated within five days of co-cultivation, with the exception of EcColinfant where a small subpopulation of *Y*. *enterocolitica* persisted. Two-tailed Student’s t-test was used for statistical comparisons (*p < 0.05, **p < 0.01, ***p < 0.001). Data were obtained from three independent experiments. The detection limit of the method was 25 CFU/ml. The microphotographs show inhibition zones resulting from probiotic colicin F_Y_ production on agar plates with susceptible *Y*. *enterocolitica*; isogenic colicin F_Y_ nonproducers did not form inhibition zones (except for a presence of a weak halo around EcColifant). The numbers of recombinant colicinogenic *E*. *coli* strains during co-cultivation are shown in Supplementary Fig. S3.

The activity of recombinant colicin producers and isogenic colicin F_Y_-nonproducing controls against *Y*. *enterocolitica* was tested on agar plates and also during continuous *in vitro* co-cultivation in broth (Fig. 3). On agar plates, production of colicin F_Y_ resulted in inhibition of *Y*. *enterocolitica*. In broth, *Y*. *enterocolitica* retained a stable concentration of approximately 10^9^ CFU/ml when grown alone, while the presence of *E*. *coli* producing colicin F_Y_ significantly reduced the numbers of pathogen after 48 h of co-cultivation (Fig. 3). Due to competition, colicin F_Y_ nonproducers were also able to decrease the numbers of pathogenic *Y*. *enterocolitica* in co-cultivation; however, production of colicin F_Y_ enhanced inhibition activity of the tested *E*. *coli* strains and the *Y*. *enterocolitica* was completely or nearly completely eliminated after five days of co-cultivation (Fig. 3). In the presence of colicin F_Y_, pathogen elimination was observed for strains EcH22, Ec1127, and Ec1145, but not for EcColinfant, where *Y*. *enterocolitica* persisted at detectable levels.

### *In vivo* activity of recombinant colicinogenic *E*. *coli* against pathogenic *Y*. *enterocolitica*

**Figure 4:**
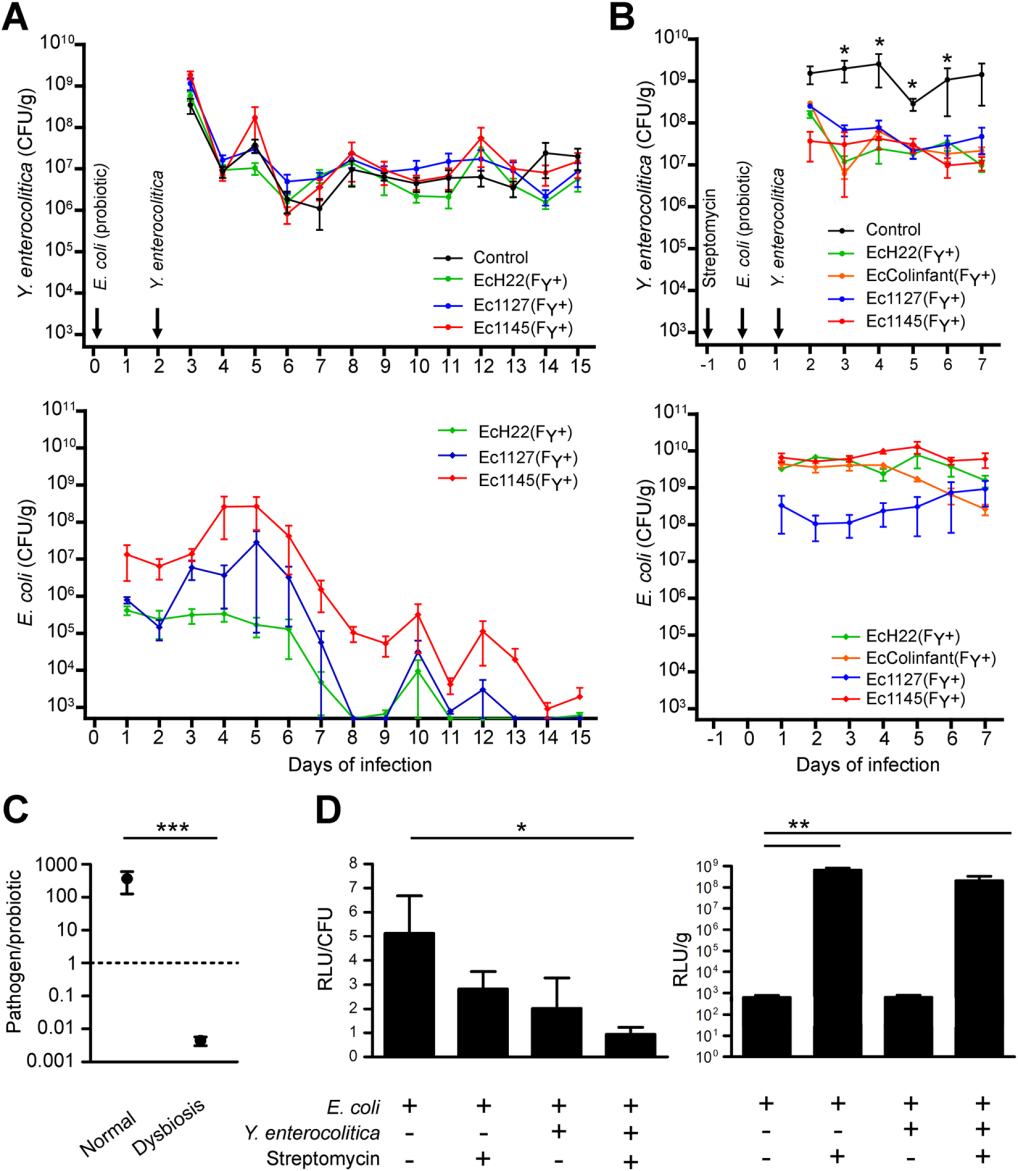
*In vivo* activity of recombinant colicin F_Y_-producing *E*. *coli* against *Y*. *enterocolitica*. (**A**) Mice with a normal microflora (n = 5; each group) were inoculated with colicin F_Y_*-*producing *E*. *coli* and then with *Y*. *enterocolitica*. Only *Y*. *enterocolitica* was administered to controls (n = 5). Numbers of shed *Y*. *enterocolitica* were counted and plotted (mean ± SEM). No statistical differences in *Y*. *enterocolitica* numbers were observed between treated and untreated animals (top). Based on the *E*. *coli* numbers, recombinant *E*. *coli* colonized the intestines transiently (bottom). Raw data are shown in Supplementary Fig. S4. (**B**) Streptomycin-treated mice (n = 5; each group) were inoculated with colicin F_Y_-producing *E*. *coli*, and then infected with *Y*. *enterocolitica*. Only *Y*. *enterocolitica* was administered to the streptomycin-treated controls (n = 3). Numbers of shed *Y*. *enterocolitica* were counted and plotted (mean ± SEM). For all the probiotics used, significant decreases of pathogen numbers were found between days D3 and D6 (top). At the same time, the *E*. *coli* numbers remained stable during the experiment (bottom). (**C**) Based on the numbers of bacteria in the first five days of infection, the ratio of *Y*. *enterocolitica* to Ec1145 in normal mice versus the streptomycin-treated mice showed that the pathogen significantly outgrew the probiotic in the normal mice, while Ec1145 dominated in the dysbiotic mice. (**D**) Mice (n = 5; each group) were treated or not with streptomycin and/or with *Y*. *enterocolitica* a day later. After another 24 h, mice were inoculated with Ec1145 expressing the luciferase under *lac*-*pcib* regulation; the colon contents were collected two days later and subjected to a luciferase assay. Reporter expression calculated for a single bacterial cell (CFU) was within a range of one order of magnitude across the treatments (left), but the total levels of reporter were significantly increased in the intestinal dysbiosis compared to intestines with normal microflora (right). Data are presented as the mean ± SEM. RLU, relative luciferase unit per second. (**A**–**D**) Two-tailed Mann–Whitney–U test was used for statistical comparisons (*p < 0.05, **p < 0.01, ***p < 0.001).

Colicin F_Y_ activity was tested *in vivo* under several experimental settings. First, colicin F_Y_ activity was tested in mice with normal microflora. Since EcColinfant showed low colonization capacity and had weak inhibition during co-cultivation, only the remaining three colicin F_Y_*-*producing *E*. *coli* strains were used during experimental *Y*. *enterocolitica* infection of mice. Recombinant *E*. *coli* was applied to experimental animals via drinking water and after 48 hours, animals were infected with pathogenic *Y*. *enterocolitica* in the same way. Clinical manifestations and the number of pathogenic *Yersinia* (and also recombinant *E*. *coli*) in the feces were monitored daily; both parameters stayed unaffected during the 15 days of the experiment (Figs 4A and S4). The use of different applications, inoculation doses, administration times, and application of various recombinant *E*. *coli* strains had no effect on *Y*. *enterocolitica* (Supplementary Fig. S4).

Second, the *in vivo* activity of colicin F_Y_ was tested using a mouse model, in which streptomycin was used to decrease the gut microflora and to promote gut inflammation^26^. Four recombinant *E*. *coli* (including EcColinfant) strains with colicin F_Y_ expression regulated by *lac*-*pcib* promoters were used during experimental *Y*. *enterocolitica* infection of streptomycin-treated mice. Twenty-four hours after streptomycin application, recombinant colicinogenic *E*. *coli* was applied to experimental animals via drinking water, and after another 24hours, the animals were infected with pathogenic *Y*. *enterocolitica* in the same way. The clinical manifestation and number of pathogenic *Yersinia* (and also recombinant *E*. *coli*) in feces were monitored daily. *Y*. *enterocolitica* infection was decreased by the presence of colicinogenic *E*. *coli* in the gastrointestinal tract of streptomycin-treated mice by one to two orders of magnitude (*p < 0.05; Fig. 4B). The levels of all tested recombinant *E*. *coli* strains remained stable throughout the experiments (Fig. 4B). Based on the pathogen-to-probiotic ratio, the relative amount of probiotic *E*. *coli* was elevated by almost 6-orders in the streptomycin model compared to mice with normal gut microflora (***p < 0.001; Fig. 4C). In addition, promoter activity regulating colicin F_Y_ expression in mice intestines was analyzed using the luciferase assay. While the reporter gene expression by a single bacterium was similar (i.e., difference between signals within one order of magnitude) in mice with normal and streptomycin-treated microflora, the total reporter signal in the colon contents was raised by several orders of magnitude in the streptomycin-treated mice (**p < 0.01; Fig. 4D). Taken together, intestinal dysbiosis increased the colonization capacity and allowed *in vivo* activity of the colicinogenic *E*. *coli* against the pathogenic *Y*. *enterocolitica*.

### *In vivo* activity of colicin F_Y_ using recombinant isogenic *Y*. *enterocolitica* strains

**Figure 5:**
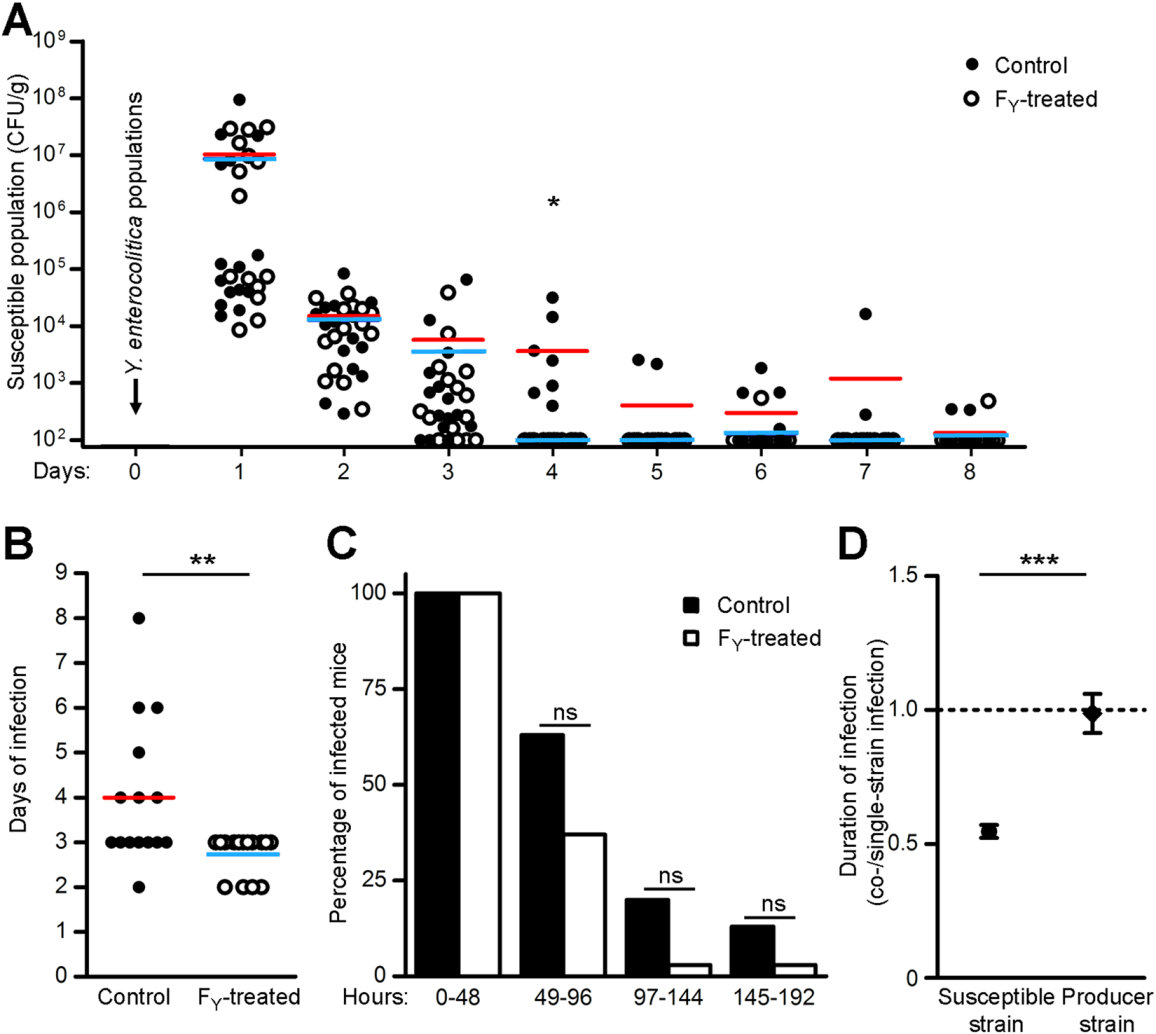
Colicin F_Y_ activity in mice with normal gut microflora using two isogenic populations of *Y*. *enterocolitica*. Fifteen mice were simultaneously inoculated with two recombinant isogenic *Y*. *enterocolitica* strains, one a colicin F_Y_ producer and the other a colicin-susceptible indicator. In the control group (n = 15), only the colicin-susceptible *Y*. *enterocolitica* indicator strain was administered. Numbers of shed *Y*. *enterocolitica* were counted, plotted and two-tailed Mann–Whitney–U test was used for statistical comparisons (*p < 0.05, **p < 0.01). Compared to the control group (black dot; red bar, mean), animals treated with the colicin-producer (white dot; blue bar, mean) showed lower numbers of colicin-susceptible *Y*. *enterocolitica* (**A**) and a shorter duration of infection (**B**). (**C**) In the presence of a colicin F_Y_ producer, mice showed a tendency towards less frequency of susceptible *Y*. *enterocolitica* infection than the control animals (Fisher’s exact test). ns, not significant. (**D**) The ratio of the co-infection period to the single infection period for the susceptible strain and for the producer strain. While susceptible *Y*. *enterocolitica* infection was shorter in the presence of colicin F_Y_ than in controls, the shedding period of colicin-producing *Y*. *enterocolitica* was not affected. Data are presented as the mean ± SEM and two-tailed Mann–Whitney–U test was used for statistical comparison (***p < 0.001). Detection limit of the method was 100 CFU/g of feces (**A**). The end of infection was defined as two consecutive days without the pathogen being detected (**B**,**D**).

Besides the colonization resistance shown by intestines with normal microflora, the activity of recombinant *E*. *coli* against *Y*. *enterocolitica* could also be limited by spatial differences in their intestinal niches, i.e., avoiding their direct interaction. Therefore, two isogenic strains of *Y*. *enterocolitica*, a colicin producer and a colicin-susceptible indicator, were constructed (see Methods) and tested in mice with normal gut microflora using simultaneous administration. Compared to the control group (without a colicin producer), administration of colicin-producing *Y*. *enterocolitica* limited the numbers of the colicin-susceptible strain (*p < 0.05 on day four; Fig. 5A), shortened the infection period of the susceptible strain (**p < 0.01; Fig. 5B), and showed a tendency towards lower numbers of infected animals with the susceptible strain (Fig. 5C). While the infection period of susceptible *Y*. *enterocolitica* was decreased in the presence of colicin F_Y_, the infection period of the colicin producer was not affected (Fig. 5D); thus, the infection period limitation of colicin-susceptible strain was not due to co-cultivation.

## Discussion

In this study, recombinant probiotic *E*. *coli* strains producing colicin F_Y_ were shown to inhibit *Y*. *enterocolitica in vitro* and *in vivo*. Besides colicin F_Y_^20^, pesticin I and enterocoliticin were characterized at various levels of detail and both showed activity against pathogenic yersiniae *in vitro*^29,30^; however, their activity was not clearly confirmed *in vivo*^31^. As shown in a previous study, colicin F_Y_ inhibited all tested *Y*. *enterocolitica* isolates belonging to the most common serotypes^21^ and therefore production of colicin F_Y_ was predicted as a promising feature of probiotic strains in the treatment of gastrointestinal yersiniosis^21^. To date, production of colicin F_Y_ had not been identified in *E*. *coli* isolates; therefore, recombinant *E*. *coli* strains producing colicin F_Y_ had to be constructed for this study.

Out of four *E*. *coli* strains used, probiotic features were previously ascribed to EcH22 and EcColinfant. The human probiotic EcH22 was shown to produce microcin C7 (and several other antimicrobial substances), which mediated an inhibition of pathogenic *Shigella* in the gnotobiotic mice model^28^. The EcColinfant strain is a component of the probiotic product “Colinfant New Born” marketed by Dyntec (Terezín, Czech Republic). In the Czech Republic, it is used for several conditions in newborns and infants, such as disorders in the composition of intestinal microflora after antibiotic treatment (reviewed in^12^). Since human *E*. *coli* strains were shown to be weak colonizers of the murine gut^26,27^, two additional strains, Ec1127 and Ec1145, were isolated from mice and used in this study. The mice model used to study of colicin F_Y_ activity against pathogenic *Yersinia* is well-established model for human yersiniosis^32,33^.

As shown in this study, recombinant *E*. *coli* producing F_Y_ eliminated the growth of *Y*. *enterocolitica* serotype 0:3 during *in vitro* cultivation over five days. In general, production of bacteriocins is regarded as an important feature for probiotic efficacy^13^ and several theoretical studies have suggested that bacteriocin producers have a competitive advantage over non-producers when colonizing the same ecological niche^34–36^. In fact, activity of bacteriocins against other (pathogenic) bacteria has been described in many experimental *in vitro* studies, but bacteriocin activity in animal models is not well established (reviewed in^13,37,38^). Studies of colicin activity *in vivo* often show no or only subtle effects of colicin synthesis^35,39,40^. Inactivation of colicins by intestinal proteases or reduced colicin activity under anaerobic conditions have been suggested as the underlying reasons for their *in vivo* inactivity^41,42^. Using the luciferase assay, we showed that colicin F_Y_ recombinant promoters (i.e., *lac-pcib*) were active in mice guts and moreover, colicin F_Y_ stayed fully active for more than forty-five minutes when cultivated with the contents of the murine colon.

Despite expression of colicin F_Y_ and its intestinal stability, together with *in vitro* activity, the *in vivo* activity of colicin F_Y_ against *Y*. *enterocolitica* using mice with normal gut microflora was not observed. On the other hand, recombinant *E*. *coli* producing colicin F_Y_ inhibited *Y*. *enterocolitica* in streptomycin-treated mice. Gut inflammation, often associated with dysbiosis and expansion of *Enterobacteriaceae*, results in stress conditions including induction of the SOS-response and iron limitation^43–45^. Expression of colicins is tightly regulated by the SOS-response promoter, and in addition, colicins Ia, Ib, and F_Y_ are also regulated by the iron-dependent Fur promoter^20,46,47^. Recently, several studies showed that bacteriocin activity is associated with inflammatory conditions in the gut^27,43,47^. Using streptomycin-treated mice (mouse *Salmonella*-colitis model), Deriu *et al*.^43^ showed that the probiotic strain *E*. *coli* Nissle 1917, producing bacteriocins mM and mH47, outcompeted and reduced *S*. Typhimurium colonization; and the probiotic activity depended on iron acquisition by *E*. *coli* Nissle 1917. Sassone-Corsi *et al*.^27^ demonstrated that microcins H47 and M enabled the probiotic *E*. *coli* Nissle 1917 to limit the expansion of competing enterobacteria (including pathogenic *S*. Typhimurium) during intestinal inflammation using the mouse colitis model. Another study analyzed competition between colicin Ib-producing *Salmonella* Typhimurium and colicin-susceptible *E*. *coli* and revealed that gut inflammation promotes the effects of colicin Ib via the iron-dependent Fur promoter, which increases colicin production and also expression of its cognate receptor CirA, which mediates susceptibility of the competitor^47^.

While colicin F_Y_ expression was shown to be *in vitro* inducible (up to approximately ten-times) by iron limitation and the SOS-response via the gut inflammation-dependent promoter of colicin Ib (Fig. 2), the luciferase assay of recombinant *E*. *coli* strains present in the mice colon contents showed that the expression level from a single bacterial cell (RLU/CFU) was similar for mice with normal gut microflora, for streptomycin-treated mice, and for the yersiniosis model (3 days post infection of pathogen). Moreover, the expression per cell was lower in the yersiniosis model than in healthy mice (Fig. 4D), while Nedialkova *et al*.^47^ showed enhanced *in vivo* expression from the colicin Ib promotor (*pcib*) using the *Salmonella*-colitis model. These data could indicate no or subtle inflammation caused by streptomycin or yersiniosis in animal models (contrary to the *Salmonella*-colitis model^48^). In contrast, the total amount of reporter signal (RLU/g) was significantly increased (approximately 1,000,000-times) in streptomycin-treated mice (Fig. 4D). These data are consistent with a scenario in which gut inflammation in the streptomycin/yersiniosis model is not sufficient to upregulate colicin F_Y_ synthesis; however, the colonization capacity of recombinant colicinogenic *E*. *coli* is significantly enhanced as a result of dysbiosis (i.e., elimination of gram negative bacteria) in streptomycin-treated mice.

In this study, recombinant colicinogenic *E*. *coli* colonized the gut of normal mice with yersiniosis, but only transiently, while streptomycin-treatment allowed a robust, stable, and long-term *E*. *coli* colonization of the mouse gut (Fig. 4). The increased number of *E*. *coli* found in streptomycin-treated mice suggests that colonization resistance is the major limitation of the use of mice with a normal gut microflora. Colonization resistance is defined as an inhibition of invading microorganisms by resident microflora during healthy homeostasis^49^. Streptomycin treatment reduces colonization resistance and opens the *E*. *coli* niches, which are occupied by the resident strains during homeostasis.

To avoid the effect of *E*. *coli* colonization resistance and to show *in vivo* colicin F_Y_ activity, an alternative approach was used. Since colonization resistance was not observed for pathogenic *Y*. *enterocolitica*, a model of two isogenic *Y*. *enterocolitica* populations was used to study colicin F_Y_ in mice with normal microflora. In this model, colicin F_Y_-producing *Y*. *enterocolitica* inhibited colicin-susceptible *Y*. *enterocolitica* (Fig. 5) and thus, *in vivo* colicin F_Y_ activity was, for the first time, demonstrated using animals with normal intestinal microflora.

In conclusion, the activity of colicin F_Y_ *in vivo* was clearly demonstrated; however, the use of probiotic *E*. *coli* strains synthesizing colicin F_Y_ in the normal mice model was not successful since the active colicin F_Y_ molecule was not delivered to the target *Y*. *enterocolitica* cells, most likely because of a combination of colonization resistance and spatially different intestinal niches. Besides attenuated/nonpathogenic *Yersinia*, other candidates for delivery of colicin F_Y_ to target could be tested among the relatively abundant classes of *Firmicutes* or *Bacteroidetes* (e.g., *Lactococcus*, *Lactobacillus*, and *Pediococcus*^50–55^). Alternatively, sufficient *in vivo* quantities of the active molecule could be ensured by direct application of the purified bacteriocin^56,57^.

Moreover, this study demonstrated that colicin F_Y_ synthesis could be an important feature of probiotic *E*. *coli* strains. Although the effect of probiotic *E*. *coli* strains is limited under healthy conditions, the potential effect of probiotic *E*. *coli* strains and synthesized bacteriocin molecules appear to be more effective under dysbiotic conditions in the gut. Taken together, colicin F_Y_ itself appears to have sufficient activity for treatment of gastrointestinal yersiniosis, however, the suitable application form needs to be experimentally determined.

## Methods

### Bacterial strains and growth conditions

*Yersinia frederiksenii* strain Y27601 producing colicin F_Y_^20^, two pathogenic *Yersinia enterocolitica* strains (serotypes O:3 and O:8)^58^, and two *Escherichia coli* strains with probiotic features including *E*. *coli* O83:K24:H31 (EcColinfant; isolated from “Colinfant New Born”) and *E*. *coli* H22 (EcH22)^28^, were obtained from our laboratory stock. Two murine *E*. *coli* strains (i.e., Ec1127 and Ec1145) were isolated and characterized in this study (see below). The list of strains and plasmids constructed in this work is shown in Supplementary Table S1.

Tryptone-yeast (TY) broth consisting of 8 g/l tryptone (Hi-Media), 5 g/l yeast extract (Hi-Media), and 5 g/l sodium chloride in water was used throughout the study. For cultivation on solid media, TY broth was supplemented with agar powder (1.2%, w/v; Hi-Media). TY agar plates supplemented with chloramphenicol (final concentration 0.025 g/l; Sigma-Aldrich) or kanamycin (0.050 g/l; Sigma-Aldrich) were used for selection and maintenance of recombinant strains. Pathogenic *Y*. *enterocolitica* was cultivated on plates with selective diagnostic CIN agar (Cefsulodin-Irgasan-Novobiocin; Hi-Media). Streptomycin-resistant variants of strains used in this work were selected by cultivation on agar plates supplemented with streptomycin (0.050 g/l; Sigma-Aldrich). The cultivations of *E*. *coli* and *Y*. *enterocolitica* strains were performed at 37 °C and 30 °C, respectively.

### Identification and characterization of murine *E*. *coli* isolates

For five days, the feces from four healthy control BALB/c mice and five BALB/c mice with experimental yersiniosis and stably shedding *Y*. *enterocolitica* (for twenty days) were collected. All feces were diluted in PBS, homogenized, and spread on selective diagnostic Endo agar (Hi-Media). After cultivation (overnight, 37 °C), three colonies were picked from each plate and taxonomically identified using ENTEROtest16 (Erba Lachema, Brno, Czech Republic). All 127 identified murine *E*. *coli* isolates were analyzed using *XbaI* digestion of genomic DNA and pulsed field gel electrophoresis (PFGE; PulseNet protocol (CDC 2002))^59^. PFGE profiles were analyzed using BioNumerics fingerprinting software (Applied Math).

For *in vivo* colonization capacity, animal experiments were performed by a licensed staff at an accredited facility of the Veterinary Research Institute (Brno, Czech Republic). Female BALB/c mice (6–9 weeks old) were kept individually in conditions without the presence of specific pathogens. Each experimental group contained five mice, which were orally infected with a single dose of recombinant *E*. *coli* strain (10^7^ CFU using a gastric probe). During 15 days, fresh feces from each mouse were collected daily, homogenized in PBS, 10-fold serially diluted, and spread on agar plates with kanamycin. Numbers (CFU/g feces) of shed recombinant *E*. *coli* were calculated.

### Construction of recombinant strains

Two recombinant plasmids harbouring colicin F_Y_ locus were used in this study including the pDS1006 with colicin F_Y_ expression under the control of *lac* promoter and the pDS1281 with colicin F_Y_ expression under the control of *lac-pcib* promoters. pDS1006 was described previously^20^; briefly, colicin F_Y_ activity and immunity genes (*cfyA* and *cfyI*, respectively) from the original producer *Y*. *frederiksenii* Y27601 was cloned into pCR2.1-TOPO vector using a TOPO TA Cloning Kit (Invitrogen). The pDS1281 is a modification of the pDS1006 that harbors additional colicin Ib promoter (*pcib*)^47^ and it was constructed in this study using an In-Fusion HD Cloning kit (Clontech). Briefly, the *pcib* (200 nt in length) was amplified from genomic DNA of *E*. *coli* 360/79 (i.e., from the original producer of colicin Ib) using *Pfu* polymerase (Fermentas) and specific primers with overlaps complementary to the pDS1006 (Supplementary Table S1). The *pcib* amplicon was cloned (via recombination) into pDS1006 upstream of colicin F_Y_ gene, between *lac* promoter and colicin F_Y_ gene. Both plasmids, pDS1006 and pDS1281, were transformed into strains EcH22, EcColinfant, Ec1127, and Ec1145, which resulted in 8 various recombinant *E*. *coli* strains, all producing colicin F_Y_.

To test the colicin F_Y_ recombinant expression, the pJB008 harboring a reporter gene (i.e, a firefly luciferase; *luc*) downstream of *lac-pcib* promoters was constructed. Briefly, *luc* gene was amplified from the vector pGL4.17 (Promega) using *Pfu* polymerase (Fermentas) and specific primers with overlaps complementary to the pDS1281 (Supplementary Table S1). Then, colicin F_Y_ gene in the pDS1281 was replaced by *luc* gene (via recombination) using E. cloni® 10G (Lucigen Corporation).

For construction of recombinant isogenic *Y*. *enterocolitica* strains, a commercial vector, pBeloBAC11 (New England Biolabs), encoding chloramphenicol resistance was electro-transformed into the colicin F_Y_-susceptible indicator *Y*. *enterocolitica* strain 8081. Alternatively, a modified suicide vector pNKBOR^60^ carrying the recombinant colicinogenic locus *pcib*-*cfy*A-*cfy*I was constructed (pJB001) and used for stable insertion of the recombinant colicinogenic locus into the *Y*. *enterocolitica* 8081 chromosome, resulting in the *Y*. *enterocolitica cit*E::*cfy*A-*cfy*I strain. The insertion was mapped to the *cit*E gene (YE2651; NC_008800.1^61^, position 2,868,244) and this insertion did not significantly affect the *in vitro* grown of mutant strain (Fig. S5).

Streptomycin-resistant variants of selected bacterial strains were obtained by cultivation in the presence of streptomycin (see above). A spontaneous mutation in the *rpsL* gene (position 248T) was identified by sequencing.

### Analysis of colicin F_Y_ recombinant expression

Crude colicin F_Y_ extract was prepared as previously described^20^. Briefly, a 20-fold-diluted overnight TY culture of a colicinogenic strain was cultivated (37 °C, 200 rpm, 4 h), induced by mitomycin C (final concentration 500 µg/l) or iron limitation (0.2 mM 2,2′-dipyridyl and 0.1 mM Nitrilotriacetic acid trisodium salt), cultivated for additional 4 h (37 °C, 200 rpm), and centrifuged (15 min, 4,000 × *g*). The bacterial pellet was resuspended in 5 ml of distilled water, washed twice, and sonicated. The resulting bacterial lysate was centrifuged for 15 min at 4,000 × *g*, and the supernatant was used as a crude colicin F_Y_ extract. Colicin activity was tested by spotting 2-fold serial dilutions of colicin extract on agar plates with a thin layer of 0.75% agar containing a colicin-susceptible *Yersinia* strain (inoculated with 10^8^ cells). After overnight incubation of plates at 37 °C, the inhibition zones of susceptible *Yersinia* were determined. The highest dilution of colicin suspension causing growth inhibition represented expression of colicin F_Y_.

### Analysis of colicin stability in the gastrointestinal tract

Female BALB/c mice (ca. 20 weeks old) were kept in conditions without the presence of specific pathogens. After cervical dislocation, mice intestinal contents from the stomach, ileum, cecum, and colon were separately collected in four fractions. For each intestine fraction, content was homogenized in PBS buffer (1 ml) by pippeting, centrifuged briefly (1 min, 14,000 × *g*), and supernatants were stored at −20 °C.

For analysis of colicin F_Y_ stability under gastrointestinal tract conditions, crude colicin F_Y_ extract (see above) was mixed with isolated fractions of intestinal contents (volume 1:1), incubated at 37 °C, and stopped at various time-points (i.e., at 0, 4, 8, 10, 20, 30, 45, 60, and 120 minutes) using a protease inhibitor cocktail (cOmplete™; Roche). Residual colicin activity was tested by spotting 10-fold serial dilutions of suspensions on agar plates with a colicin-susceptible *Yersinia* strain. After overnight incubation, the highest dilution of suspension causing growth inhibition of *Yersinia* was determined.

### Analysis of colicin activity *in vitro*

Overnight cultures of *Y*. *enterocolitica* Y11 (30 °C, 200 rpm) and colicin F_Y_ producer (kanamycin, 37 °C, 200 rpm) were separately cultivated overnight in TY broth. Then, they were mixed (100 µl each) in fresh TY broth (5 ml) and were co-cultivated (37 °C, 200 rpm). The mixed bacterial suspension (100 µl) was inoculated daily into fresh TY broth (5 ml) for 15 days. In addition, aliquots of the bacterial suspension were collected and 10-fold serial PBS dilutions were spread on selective agar plates (i.e., kanamycin plates for colicin producer and CIN plates for *Y*. *enterocolitica*). Plates were cultivated overnight (37 °C) and bacterial numbers were counted.

### Analysis of colicin activity *in vivo*

Animal experiments and handling were performed by a licensed staff at an accredited facility of the Veterinary Research Institute (Brno, Czech Republic). Female BALB/c mice (6–9 weeks old) were kept in conditions without the presence of specific pathogens. In the experiment, each group contained at least five experimental mice and each mouse was kept in the individual cage. Mice were orally infected (10^8^ CFU/ml of the drinking water) with pathogenic *Y*. *enterocolitica* Y11. Recombinant strains producing colicin F_Y_ were also orally administered (10^8^ CFU/ml) to experimental animals. Mice were monitored for weight, for condition of feces, and for clinical manifestation of yersiniosis. In addition, fresh feces were collected daily and processed, within 2 hours, for microbiological analysis. Fecal sample from each mouse was homogenized in PBS, 10-fold serially diluted, and spread on selective agar plates. While *Y*. *enterocolitica* was cultivated on CIN agar, recombinant *E*. *coli* producing colicin F_Y_ were selected on agar plates with kanamycin. Numbers (CFU/g feces) of pathogenic *Y*. *enterocolitica* and colicin-producers were calculated. In the streptomycin-treated mice model, streptomycin (5 g/l) was added to the drinking water 24 hours before inoculation and was applied during the whole experiment.

### Analysis of colicin *in vivo* expression using luciferase assay

Luciferase assays were performed using a Luciferase assay system (E1500; Promega) according to the manufacturer’s instructions and as previously described Nedialkova *et al*.^47^. Briefly, the mice (i.e., conventional mice, streptomycin-treated mice, and mice infected by *Y*. *enterocolitica* Y11; 5 mice per group) were inoculated with Ec1145 expressing a luciferase reporter (pJB008). Two days after application of reporter Ec1145, the colon contents were aseptically harvested from mice, thoroughly suspended in PBS (1,000 µl), and filtered through a 40 µm cell filter (Corning Cell Strainer). A defined volume of colon suspension (500 µl) was pelleted (4 °C, 10 min, 14,000 × *g*), the pellet was suspended in 1 M K_2_HPO_4_/20 mM EDTA (10 µl), frozen on dry ice (1 min), and stored at −80 °C. After thawing at room temperature, suspensions were mixed with 300 µl of fresh lysis buffer (1xCell Culture Lysis Reagent [E1531, Promega], 1,25 mg/ml lysozyme, and 2,5 mg/ml BSA), incubated (10 min, RT), and bacterial lysates (10 µl) were transferred into 96-well plates (3922; Costar). Using a TriStar^2^ LB 942 Modular Multimode Microplate Reader (Berthold Technologies), luciferase reagent was added to each well (40 µl), luminescence was measured, and relative light units per second (RLU) were calculated. Only values above the detection limit (control caecum contents) were considered. In addition, aliquots (50 µl) of colon suspensions were 10-fold serially diluted in PBS and spread on selective agar plates. After overnight cultivation, the amount (CFU) of reporter strain was determined and RLU per CFU of reporter strain was calculated.

### Statistical analysis

Prism 5 software (GraphPad) was used for statistical analyses. Bacterial growth during *in vitro* experiments was analyzed using the unpaired Student’s *t*-test. The non-parametric Mann–Whitney–U test was used to analyze bacterial CFUs in feces, RLU/CFU in colon contents, and infection length. In addition, Fisher’s exact test was used for analysis of the frequency of yersiniosis. P-values less than 0.05 (2-tailed) were considered statistically significant, and were denoted with asterisks (*p < 0.05, **p < 0.01, and ***p < 0.001).

### Ethical approval and informed consent

The experimental protocol was approved by the Branch Commission for Animal Welfare of the Ministry of Agriculture of the Czech Republic (permission 22019/2016-MZe-17214) in accordance with Act No. 246/1992 Coll., on the protection of animals against torture, as subsequently amended, and with Decree 419/2012 Coll. on the protection, breeding and use of experimental animals.

## Electronic supplementary material

Fig S1, Fig S2, Fig S3, Fig S4, Fig S5, Table S1

## Data Availability

All relevant data are within the paper and its Supporting Information files.
