# Supplementary material for: Colicin FY inhibits pathogenic Yersinia enterocolitica in mice
Source: Sci Rep. 2018 Aug 16;8:12242. doi: 10.1038/s41598-018-30729-7 (PMC6095899; doi:10.1038/s41598-018-30729-7)
Supplement: Supplementary file 1 — Fig S1, Fig S2, Fig S3, Fig S4, Fig S5, Table S1 [file 41598_2018_30729_MOESM1_ESM.pdf]

Supplementary Information

Colicin F_Y inhibits pathogenic *Yersinia enterocolitica* in mice

Juraj Bosák[1], Lenka Micenková[2], Matěj Hrala[1], Katarína Pomorská[1], Michaela Kunová Bosáková[1], Pavel Krejčí[1], Eduard Göpfert[3], Martin Faldyna[3], David Šmajs[1,*]

[1]Department of Biology, Faculty of Medicine, Masaryk University, Brno, Czech Republic

[2]Research Centre for Toxic Compounds in the Environment, Faculty of Science, Masaryk, University, Brno, Czech Republic

[3]Veterinary Research Institute, Brno, Czech Republic

*Corresponding author:

E-mail: dsmajs@med.muni.cz

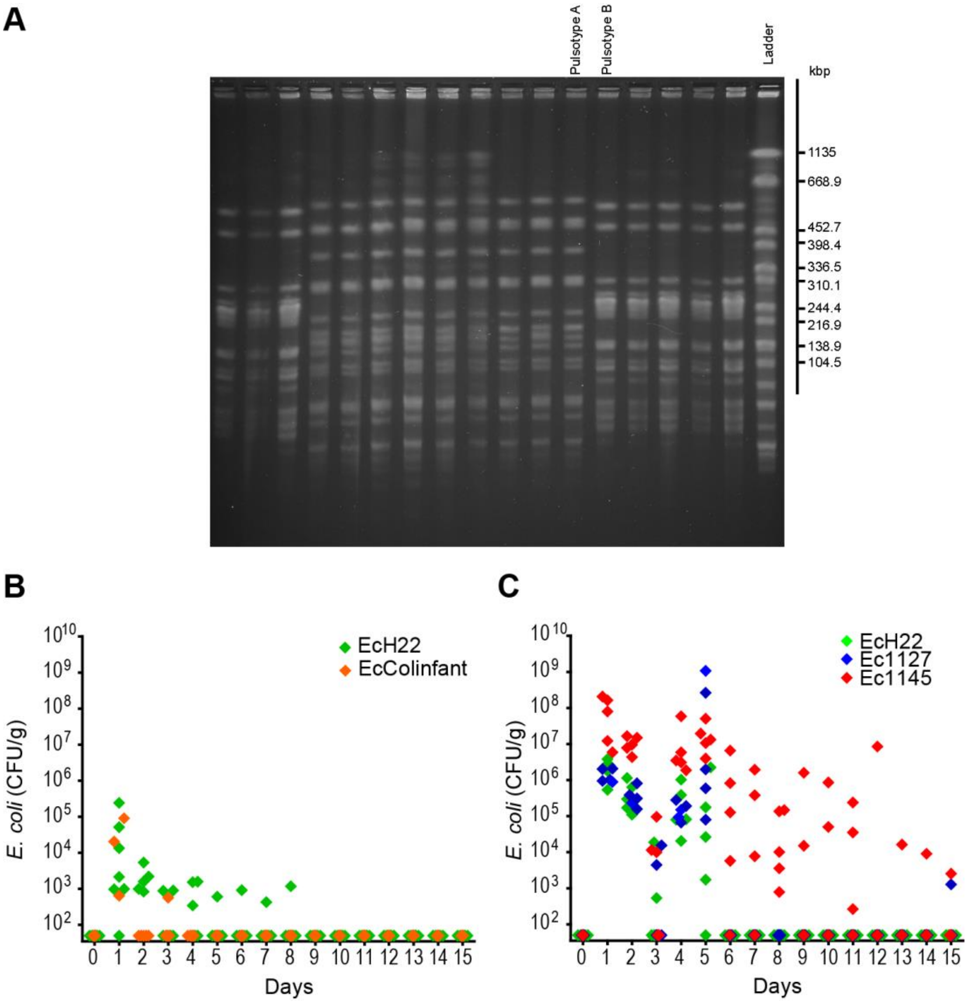

Fig S1. PFGE analysis of murine *E. coli* isolates and colonization capacity of recombinant *E. coli* strains. A) The original gel used in Fig 1. PFGE resolved two pulsotypes among *E. coli* isolates. Two pulsotypes used in Fig 1 are marked. **B-C)** Mice (n=5; each group) were inoculated with 10^7 CFU of *E. coli* and the fecal counts of bacteria were monitored for 15 days as detailed in the Methods section. The numbers of *E. coli* shed were plotted. **B)** Colonization capacity of strains EcH22 and EcColinfant. **C)** Colonization capacity of probiotic EcH22 and two murine strains, Ec1127 and Ec1145. The detection limit of the method was 50 CFU/g feces.

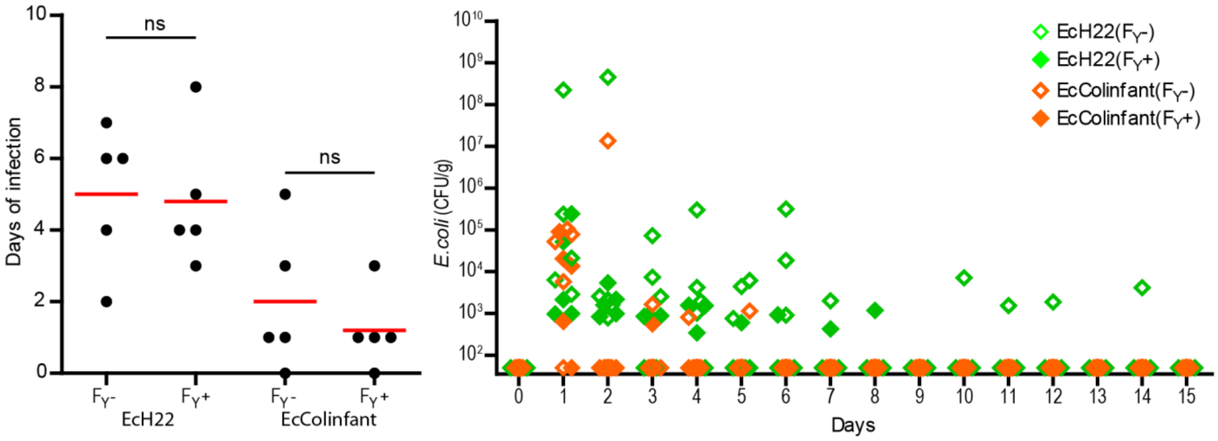

Fig S2. Colonization capacity of isogenic *E. coli* - colicin F_Y producers and nonproducers. Mice (n=5; each group) were inoculated with 10^7 CFU of *E. coli* and the fecal counts of bacteria were monitored for 15 days as detailed in the Methods section. The duration of shedding (left; red bar, mean) and the numbers of *E. coli* shed (right) were plotted. The end of colonization was defined as two consecutive days without bacteria shedding. Two-tailed Mann–Whitney–U test (*p<0.05; ns, not significant). The detection limit of the method was 50 CFU/g feces.

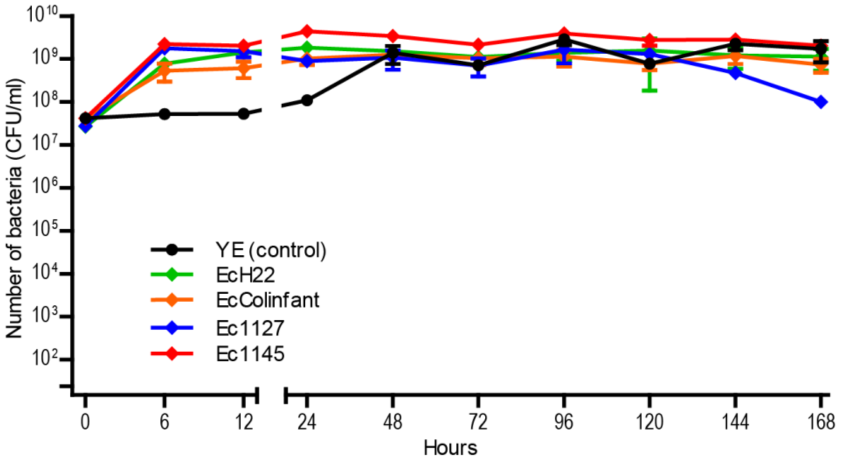

Fig S3. Numbers of *E. coli* during *in vitro* co-cultivation with *Y. enterocolitica*. Pathogenic *Y. enterocolitica* was co-cultivated with various recombinant *E. coli* strains, and the *E. coli* counts were obtained at the indicated timepoints. The data were obtained from three independent biological experiments, and were presented as mean ± SEM. Detection limit of method was 25 CFU/ml. The numbers of *Y. enterocolitica* during co-cultivation are shown in Fig 3.

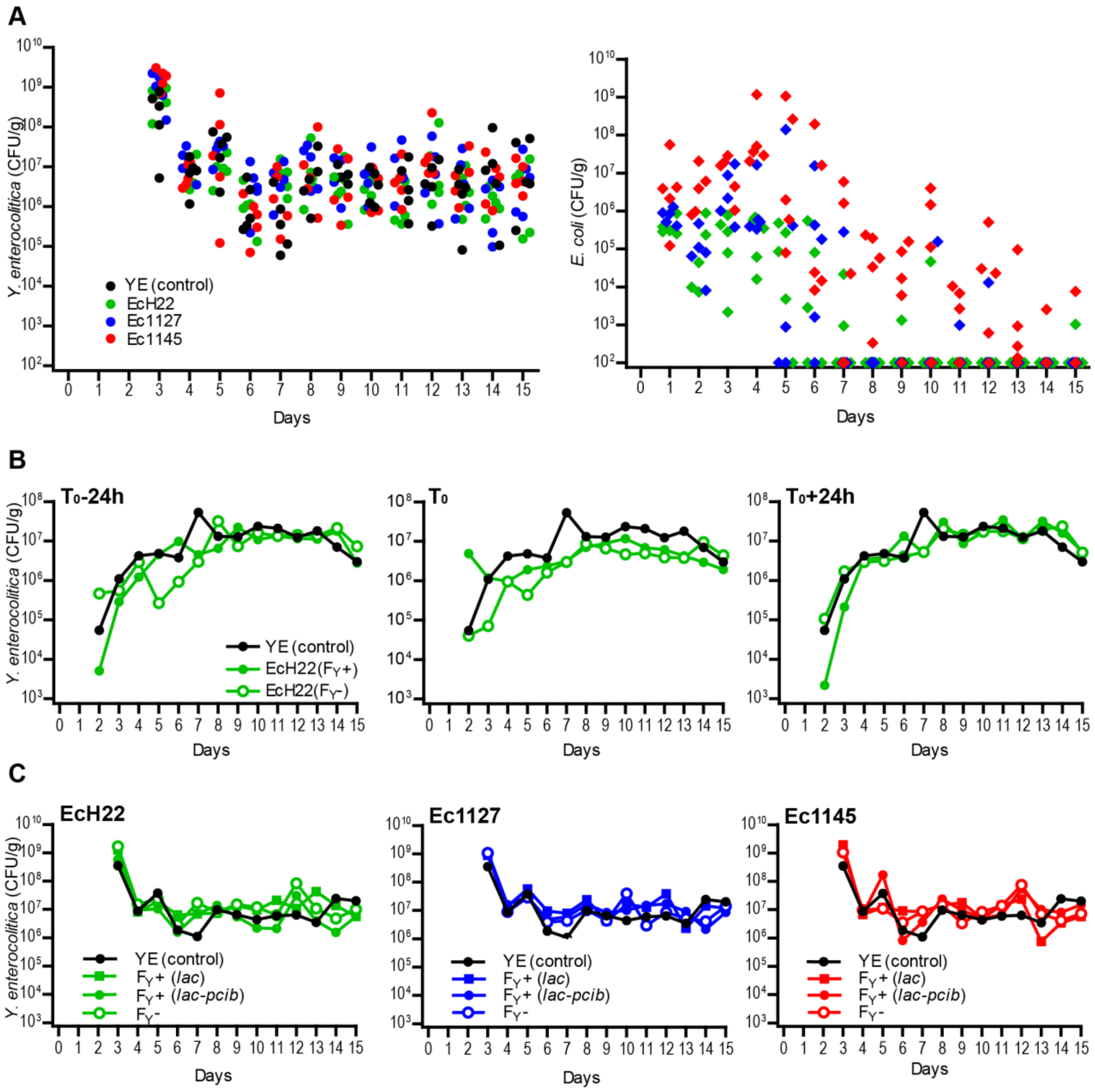

Fig S4. Activity of recombinant *E. coli* strains producing colicin F_Y against *Y. enterocolitica* using mice with normal microflora. A) Raw data obtained from the mouse model with normal microflora (see Fig 4A). Mice (n=5; each group) were inoculated with probiotic *E. coli* strains producing colicin F_Y, and then with *Y. enterocolitica* 48 hours later. Control mice were administered with *Y. enterocolitica* alone. Two-tailed Mann–Whitney–U test did not show any differences in *Y. enterocolitica* counts between the control and colicin F_Y–treated animals (left). The numbers of *E. coli* producing colicin F_Y are shown in the right panel. **B-C)** In mice with normal microflora, activity of recombinant *E. coli* producing colicin F_Y against *Y. enterocolitica* was tested using various protocols. **B)** Recombinant EcH22 was

inoculated at 10^7 CFU using a gastric probe at three different timepoints – 24 hours before *Y. enterocolitica* infection (T_0 – 24 h), simultaneously with *Y. enterocolitica* (T_0), and 24 hours after *Y. enterocolitica* (T_0 + 24 h). **C)** Twenty-four hours before *Y. enterocolitica* infection, various *E. coli* producing colicin F_Y (either *lac* or *lac-pcib* regulation) and isogenic nonproducers were inoculated with 10^8 CFU via drinking water. In all experiments, clinical manifestation and numbers of *Y. enterocolitica* in the feces were monitored daily for 15 days. After analysis using the two-tailed Mann–Whitney–U test, the different applications, inoculation doses, administration times, and application of various recombinant *E. coli* strains were found to have no effect against *Y. enterocolitica* in mice with normal microflora. The data are presented as mean ± SEM.

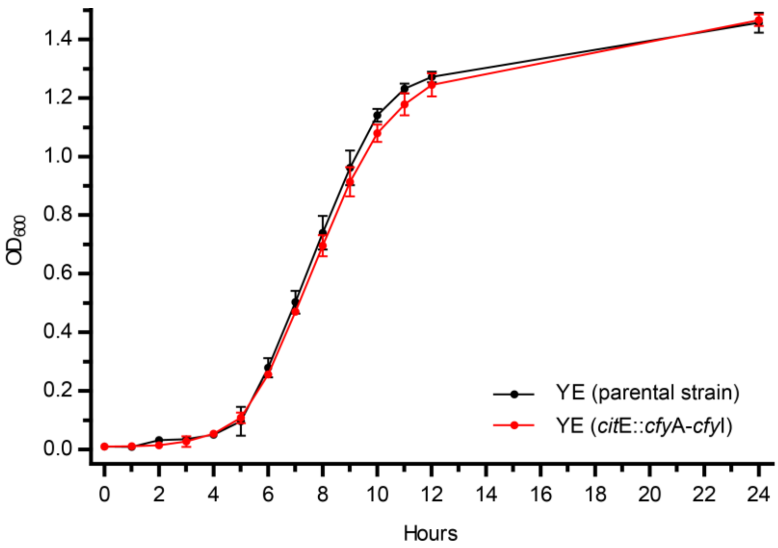

Fig S5. The growth curve of colicinogenic recombinant *Y. enterocolitica cit*E::*cfy*A-*cfy*I strain. Overnight culture (10^8 CFU) was inoculated into TY broth (100 ml) and cultivated (30 °C, 200 rpm). The optical density of bacterial suspension (OD_{600}) was measured at various time-points of cultivation. The data from three independent experiments are presented as mean ± SEM. Compare to parental strain *Y. enterocolitica* 8081, growth of *Y. enterocolitica cit*E::*cfy*A-*cfy*I was not affected by the insertion of colicinogenic locus into chromosome.

S1 Table. List of strains, plasmids, and primers used in this study

Bacteria	Relevant characteristic	Source/Reference
Y. frederiksenii Y27601	original colicin F_Y producer	Bosák et al. 2012[20]
Y. enterocolitica Y11	subsp. *palearctica*; serotype O:3	Batzilla et al. 2011[58]
Y. enterocolitica 8081	subsp. *enterocolitica*; serotype O:8	Batzilla et al. 2011[58]
E. coli H22	probiotic isolate	Cursino et al. 2006[28]
E. coli O83:K24:H31	isolate from product „Colinfant New Born"	Dyntec; This study
E. coli 1127	isolate from BALB/c mice with yersiniosis	This study
E. coli 1145	isolate from healthy BALB/c mice	This study
E. coli 360/79	original colicin Ib producer; source of colIb promoter (*pcib*)	Laboratory stock
E. cloni ® 10G	commercial cloning strain	Lucigen Corp.
**Plasmids**	**Relevant characteristic**	**Source/Reference**
pDS1006	pCR2.1TOPO encoding colicin F_Y regulated via *lac* promoter	Bosák et al. 2012[20]
pDS1281	pCR2.1TOPO encoding colicin F_Y regulated via *lac* and *pcib* promoters	This study
pBeloBAC11	single-copy *E. coli* cloning vector	New England Biolabs
pNKBOR	suicide vector	Rossignol et al. 2001[60]
pJB001	pNKBOR encoding colicin F_Y regulated via *lac* and *pcib* promoters	This study
pGL4.17	vector encoding the firefly luciferase reporter gene	Promega
pJB008	pCR2.1TOPO encoding luciferase regulated via *lac* and *pcib* promoters	This study
**Primer name**	**Primer sequence (5→3)**	**Used for:**
Lin1006F	ATGACAGATTATAAAGATG	Construction of pDS1281
Lin1006R	AAGGGCGAATTCCAG	
IF-p-cib-F	CTGGAATTCGCCCTTCCAGAGAATACCCAGACT	
IF-p-cib-R	CTTTATAATCTGTCATTCGGTATCTCCTTGATCCT	
Lin1281-F	CGGTATCTCCTTGAT	Construction of pJB008
LinTOPO-R	CTCGAGTCCTAAGGG	
1281-fLuc-F2	CCTCAGAGGATCAAGGAGATACCGAATGGAAGATGCCAAAAACA	
TOPO-fLuc-R	AATTCGCCCTTAGGACTCGAGTTACACGGCGATCTTGC	
1281fy-NKBOR_KpnI-F	TCGATGCATGCCATGGTACCCCAGAGAATACCCAGACT	Construction of pJB001
immfy-NKBOR_KpnI-R	ATCGCGGCCGGGTACCATGGATATTAGATACTAT	
